# Supplementary material for: Tunable Polarons in Bose-Einstein Condensates
Source: Sci Rep. 2017 May 24;7:2355. doi: 10.1038/s41598-017-02398-5 (PMC5443808; doi:10.1038/s41598-017-02398-5)
Supplement: Supplementary file 1 — Supplementary Materials - Tunable Polarons in Bose-Einstein Condensates [file 41598_2017_2398_MOESM1_ESM.pdf]

Supplementary Materials - Tunable Polarons in Bose-Einstein Condensates

E. Compagno[1], G. De Chiara[2,*], D.G. Angelakis[3,4], and G. M. Palma[5]

[1]Department of Physics and Astronomy, University College London, Gower Street, WC1E 6BT London, United Kingdom.
[2]Centre for Theoretical Atomic, Molecular and Optical Physics Queen's University, Belfast BT7 1NN, United Kingdom.
[3]School of Electronic and Computer Engineering, Technical University of Crete, Chania, Crete, 73100 Greece.
[4]Centre for Quantum Technologies, National University of Singapore, 2 Science Drive 3, 117542 Singapore.
[5]NEST-INFM (CNR) and Dipartimento di Fisica e Chimica Università degli Studi di Palermo, Via Archirafi 36, I-90123 Palermo, Italy.
[*]Correspondence and requests for materials should be addressed to G. De Chiara (email: g.dechiara@qub.ac.uk)

Excitations of the system

In the main text the system has been analysed for zero temperature, namely in absence of excitations of the BEC mixture. However the static polaron description can be further extended even for $T\neq 0$. In this section we show that the excitation spectrum is independent from the presence of impurity atoms even for a mixture of BEC. This means that the description of our system in presence of excitations (i.e. when excitation modes are populated by temperature effects) is of static polarons and non-interacting quasi-particle excitations. This result has been proved for impurity atoms in one and two bands of a lattice, interacting with a single BEC in.[1,2]

To find the excitation spectrum we use a standard approach, namely taking into account small excitations $\hat{\zeta}_i(x)$ around the ground state of the system. Formally we operate the substitution on the BEC field operators $\hat{\Psi}_i(x)\to\psi_{0i}(x)+\theta_i(x)+\hat{\zeta}_i(x)$ up to second order terms in $\hat{\zeta}_i(x)$ in the Hamiltonian $\hat{H}=\hat{H}_a+\hat{H}_b+\hat{H}_{ab}+\hat{H}_{Ram}$, Eq. (12) and Eq. (16). First order terms are zero due to stationary conditions. Firstly we consider the case of no Raman laser coupling in Eq. (12). The excitations' Hamiltonian of a system of two BEC interacting with impurity atoms is $\hat{H}_\zeta$ where

$$
\begin{aligned}
\hat{H}_\zeta = &\sum_{i=A,B}\int dx\ \hat{\zeta}_i^\dagger(\mathbf{r})\left[H_0-\mu_i^{(b)}+g_{ii}^{(b)}4|\psi_{0i}(\mathbf{r})|^2+\sum_{i<j}g_{ij}|\psi_{0j}^2(x)|^2\right]\hat{\zeta}_i(\mathbf{r})+\\
&+\sum_{i=A,B}\int dx\ g_{ii}^{(b)}\left[\psi_{0i}^{*2}(x)\hat{\zeta}_i(\mathbf{r})\hat{\zeta}_i(\mathbf{r})+H.C.\right]+\\
&+\sum_{i<j}\int dx\ g_{ij}^{(b)}\left[\psi_{0i}(\mathbf{r})\psi_{0j}^*(\mathbf{r})\hat{\zeta}_i^\dagger(\mathbf{r})\hat{\zeta}_j(\mathbf{r})+\ \psi_{0i}(\mathbf{r})\psi_{0j}(\mathbf{r})\hat{\zeta}_i^\dagger(\mathbf{r})\hat{\zeta}_j^\dagger(\mathbf{r})+H.C.\right]
\end{aligned}
\tag{S1}
$$

We underline in particular that the latter is independent from impurity atoms and therefore the excitation spectrum is independent from impurities' presence. The Hamiltonian (S1) is a quadratic form in the operator $\hat{\zeta}_i(x)$ and therefore it can be reduced to a diagonal form $\hat{H}_\zeta=\sum_n\sum_{k\neq 0}\hbar\omega_{nk}\hat{b}_{nk}^\dagger\hat{b}_{nk}$ by Bogoliubov transformations, after rewriting it in terms of plane waves:

$$
\hat{\zeta}_i(\mathbf{r})=\frac{1}{\sqrt{V}}\sum_{\mathbf{k}}e^{i\mathbf{k}\cdot\mathbf{r}}\ \hat{c}_{ik}
\tag{S2}
$$

where we define the quasi-particle operators:

$$
\hat{b}_{nk}=\sum_i\left[u_{ni}(k)\hat{c}_{ik}+v_{ni}(k)\hat{c}_{i-k}^\dagger\right]\qquad\qquad i\in\{A,B\}
\tag{S3}
$$

as shown in.[3,4] The effect of the operators $\hat{b}_{nk}$ is to annihilate a quasiparticle which is a collective excitation of the system. The

excitation spectrum has two branches $\hbar\omega_\pm$ and explicitly, for BEC with the same density $n_{0i}{=}n/2$, it is

$$\hbar\omega_\pm(k) = \left\{ \left(\frac{\hbar^2 k^2}{2m_b}\right) \left[\left(\frac{\hbar^2 k^2}{2m_b} + (g_{AA}^{(b)} + g_{BB}^{(b)})n\right) \pm n\sqrt{\left(g_{AA}^{(b)} - g_{BB}^{(b)}\right)^2 + \left(g_{AB}^{(b)}\right)^2} \right] \right\}^{1/2} \tag{S4}$$

For little k the excitation energy depends linearly on the momentum as $\hbar\omega(k){=}v_s\hbar k{+}O(k^3)$, that means the excitations are phonon-like and v_s is the sound velocity of the BEC mixture

$$v_s^\pm = \left\{ \frac{n}{2m_b} \left[g_{AA}^{(b)} + g_{BB}^{(b)} \pm \sqrt{\left(g_{AA}^{(b)} - g_{BB}^{(b)}\right)^2 + \left(g_{AB}^{(b)}\right)^2} \right] \right\}^{1/2} \tag{S5}$$

When the Raman laser coupling is included in the system the excitation Hamiltonian (S1) becomes

$$\hat{H}_\zeta^{Ram} = \hat{H}_\zeta - \frac{\hbar\Omega}{2} \sum_{i<j} \int dx \left[\hat{\zeta}_i^\dagger(x)\hat{\zeta}_j(x) + \hat{\zeta}_j^\dagger(x)\hat{\zeta}_i(x) \right] \tag{S6}$$

Following[5] we evaluate the excitation spectrum when the Raman coupling term is included in the system. The explicit expression is quite complex and here we show it in the limit $g_{AA}^{(b)}{=}g_{BB}^{(b)}$ and $n_{0i}{=}n/2$. Explicitly

$$\hbar\omega_-(k) = \sqrt{\left(\frac{\hbar^2k^2}{2m_b} + \hbar\Omega\right)\left[\frac{\hbar^2k^2}{2m_b} + \hbar\Omega + \left(2g_{AA}^{(b)} - g_{AB}^{(b)}\right)n\right]}$$
$$\hbar\omega_+(k) = \sqrt{\frac{\hbar^2k^2}{2m_b}\left[\frac{\hbar^2k^2}{2m_b} + \left(2g_{AA}^{(b)} + g_{AB}^{(b)}\right)n\right]} \tag{S6}$$

Finally we find that the excitation spectrum in our system is unaffected by the presence of impurity atoms. We highlight that the description of our system in presence of excitations (i.e. when excitation modes are populated by temperature effects) is then of static polarons and quasi-particles with the energy spectrum in (S6).

It is interesting to analyse the excitation spectrum in the limit case of $k \to 0$, where we find that the expressions of the low excitation spectrum are

$$\hbar\omega_-(k) = \sqrt{\hbar\Omega\left(\hbar\Omega + (2g_{AA}^{(b)} - g_{AB}^{(b)})n\right)} + \frac{\hbar^2k^2}{2m_b}\left[\frac{(2g_{AA}^{(b)} - g_{AB}^{(b)})n + 2\hbar\Omega}{2\sqrt{\hbar\Omega\left(\hbar\Omega + (2g_{AA}^{(b)} - g_{AB}^{(b)})n\right)}}\right] + \mathcal{O}(k^2)$$
$$\hbar\omega_+(k) = \sqrt{\frac{(2g_{AA}^{(b)} + g_{AB}^{(b)})n}{2m_b}}\hbar k + \mathcal{O}(k^2) \tag{S7}$$

Regarding the low excitation sector from the latter we see that when the two BEC are Raman coupled the branch $\hbar\omega_-(k)$ is no longer phononic and it is characterised by an energy gap,[5] while the other is unaffected by the laser coupling term and depends only on the physical characteristics of the BEC. On the other hand, when the laser term $\hbar\Omega$ is off we see from Eq. (S6) that both branches are phononic with a different sound speed:

$$\hbar\omega_-(k) = \sqrt{\frac{(2g_{AA}^{(b)} - g_{AB}^{(b)})n}{2m_b}}\hbar k + \mathcal{O}(k^2)$$
$$\hbar\omega_-(k) = \sqrt{\frac{(2g_{AA}^{(b)} + g_{AB}^{(b)})n}{2m_b}}\hbar k + \mathcal{O}(k^2) \tag{S8}$$

References

1. M. Bruderer, A. Klein, S. R. Clark, D. Jaksch, "Transport of strong-coupling polarons in optical lattices", New J. Phys. **10**, 033015 (2008).

2. T. Yao, D. Cocks and W. Hofstetter, Phys. Rev. A **92**, 063635 (2015).

3. C. P. Search, A. G. Rojo, P. R. Berman, "Ground state and quasiparticle spectrum of a two-component Bose-Einstein condensate", Phys. Rev. A **64**, 013615 (2001).

4. E. V. Goldstein, M. G. Moore, H. Pu, and P. Meystre, "Eliminating the Mean-Field Shift in Two-Component Bose-Einstein Condensates", Phys. Lett. **85**, 5030 (2000).

5. P. Meystre, *Atom Optics*, (Springer-Verlag, New York, 2010).
